# Inaccessible mental health care is a risk factor for risky drinking behaviors among cancer survivors: an All of Us survey-based cohort study

**DOI:** 10.1007/s00520-026-11235-3

**Published:** 2026-09-17

**Authors:** Andrea Baldo, Miho Akabane, Odysseas P. Chatzipanagiotou, Gaya Spolverato, Timothy M. Pawlik

**Affiliations:** 1 Department of Surgery, The Ohio State University Wexner Medical Center, 395 W. 12 Ave., Suite 670, Columbus, OH USA; 2 Department of Surgery, Oncology and Gastroenterology, University of Padua, Padua, Italy

**Keywords:** Cancer survivors, Risky drinking behavior, Mental health, Mental health care access

## Abstract

**Purpose:**

Access to mental health care services and the impact on substance use among cancer survivors has not been well-defined. Patients with cancer may be at particular risk of mental health challenges and excessive alcohol consumption. We sought to characterize the impact of mental health care access on drinking behaviors among cancer survivors.

**Methods:**

Data on cancer survivors were obtained from the National Institutes of Health All of Us Research Program from the Basics, Overall Health, and the personal and family health history surveys. The association of access to mental health care and moderate drinking and binge drinking was assessed using multivariable logistic regression.

**Results:**

Among 18,879 cancer survivors, the majority was female (*n* = 12,160, 64.5%) and White (*n* = 14,576, 77.4%). Overall, 1934 participants (13.6%) reported exceeding moderate drinking, and 3507 (24.4%) reported binge drinking. Among participants with at least one mental health diagnosis, the most frequently reported condition was major depression (*n* = 3999, 72.9%), followed by anxiety disorder (*n* = 2648, 48.2%) and post-traumatic stress disorder (PTSD; *n* = 1113, 20.3%); 5.4% respondents reported that in the past 12 months needing mental health counseling but were unable to obtain it due to cost. Individuals who reported an unmet mental health need more often exceeded moderate drinking (21.3% vs 13.2%) or binge drank (35.3% vs 23.8%) (both *p* < 0.001). On multivariable analysis, inability to access mental health care was independently associated with exceeding moderate drinking (OR 1.48, 95% CI 1.16–1.89, *p* = 0.001) and binge drinking (OR 1.36, 95% CI 1.10–1.66, *p* = 0.004), even among participants without preexisting mental illness.

**Conclusion:**

Limited access to mental health care access was associated with an increased risk of risky drinking behaviors and excessive alcohol consumption. These findings support the integration of routine mental health screening and affordable counseling services into survivorship care pathways and highlight the need for policy efforts to close gaps in mental health care access.

**Supplementary Information:**

The online version contains supplementary material available at https://doi.org/10.1007/s00520-026-11235-3.

## Introduction

Alcohol consumption is widespread across the USA. Estimates from 2010 attributed approximately 88,000 deaths to alcohol use, as well as about $249 billion (equivalent to $2.05 per drink) in healthcare expenditures, lost productivity, and criminal justice costs [1, 2]. Alcohol consumption has been associated with several types of cancer – including cancers of the oral cavity, pharynx, larynx, esophagus, colorectum, liver, and breast with roughly 3.5% of cancer deaths in the USA attributable to alcohol use [3–5]. Alcohol consumption is further linked to an increased risk of cancer recurrence, a higher incidence of second primary malignancies, decreased efficacy of cancer treatments, and an elevated risk of complications [6–11]. Alcohol use is also highly prevalent among cancer survivors: 77.7% are current drinkers among whom 13% exceeded moderate drinking guidelines and 23.8% reported binge drinking behaviors [12].

The frequent coexistence of alcohol use and psychiatric disorders has been documented in the scientific literature [13–17]. Drinking behaviors can substantially affect the course of psychiatric conditions, worsening their progression and reducing treatment adherence [18–21]. Compared with individuals without psychiatric disorders, persons with major depressive disorder, bipolar disorder, anxiety disorder, or psychotic disorders are at a markedly higher risk of developing alcohol use disorders [15–17]. Of note, the burden of mental health problems among cancer survivors is also high. Data from one meta-analysis reported a prevalence of anxiety of 24.4% (95% CI 20.2–28.8) and depression of 23.7% (95% CI 20.1–27.4) among cancer survivors, although the heterogeneity across studies was considerable [22]. In turn, these conditions can exert a profoundly negative impact on cancer survivors, affecting overall health and increasing the risk of subsequent cancers with serious consequences for quality of life [23, 24]. Underlying mental health problems may be exacerbated by socioeconomic stressors, including family circumstances and employment challenges[25]. In this context, access to mental health care is critically important. Access to mental health services can be conceptualized across five dimensions: approachability (the ability to perceive), availability (the ability to reach), affordability (the ability to pay), acceptability (the ability to seek), and appropriateness (the ability to engage)[26, 27]. While removing barriers to mental health care is a stated priority of many national health systems, multiple obstacles to access care remain [28].

**Fig. 1:**
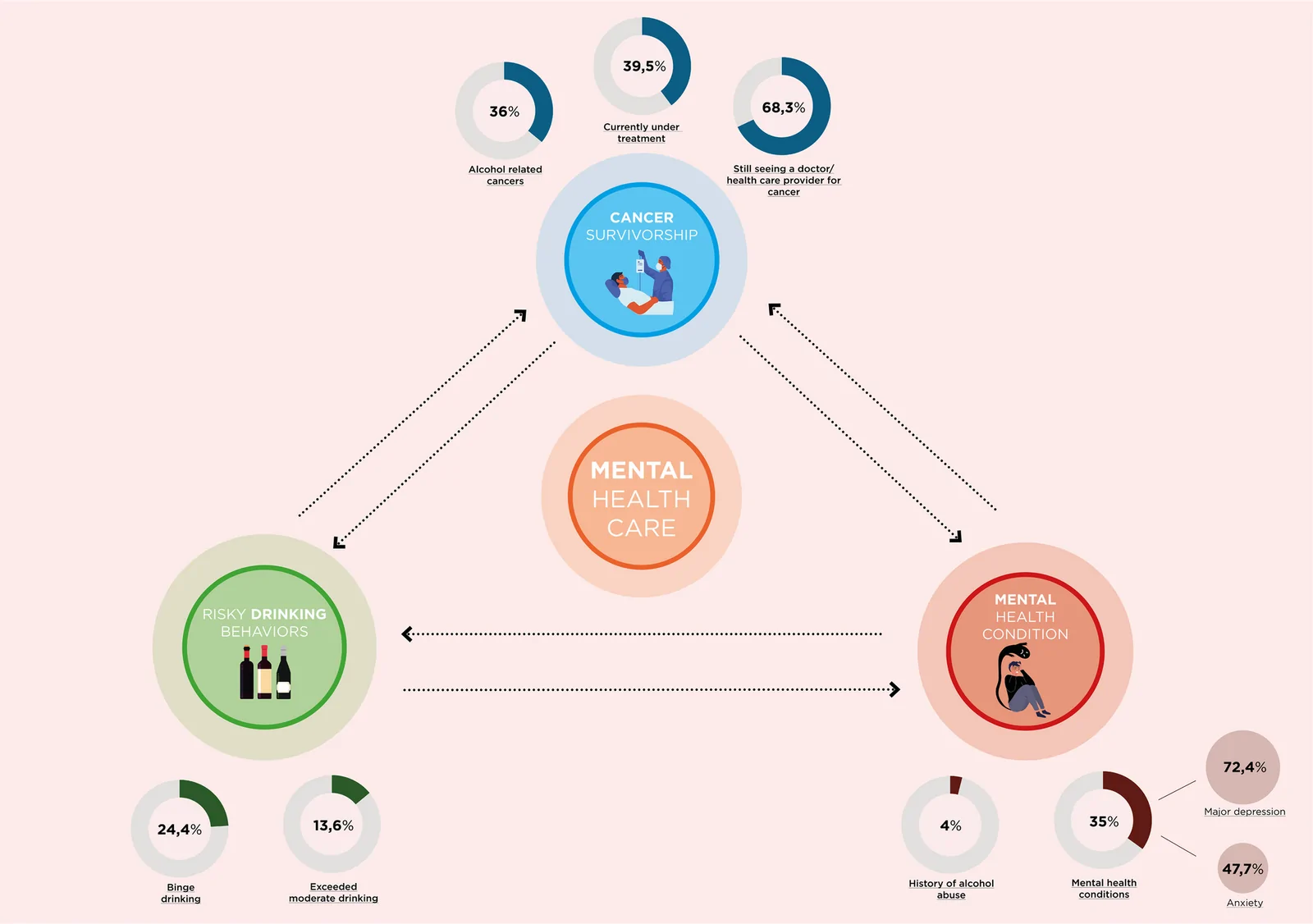
Graphic representation of the interplay between cancer survivorship, mental health and risky drinking behaviors

To date, the association of access to mental health care services and the impact on substance use among cancer survivors has not been well defined. Patients with cancer may be at particular risk of a vicious cycle of mental health challenges and alcohol consumption that affect their quality of life and survivorship. Therefore, the objective of the current study was to characterize the impact of mental health care access on drinking behaviors among cancer survivors (Fig. 1).

## Methods

### Study population

Data on cancer survivors were obtained from the National Institutes of Health All of Us Research Program. Among participants who completed the Basics, Overall Health, Lifestyle, and Personal Medical History surveys, individuals with a personal history of cancer were identified using the survey item “Including yourself, who in your family has had this cancer type?”. Only participants who selected “Self” were classified as having a personal cancer diagnosis and included in the cohort; participants who indicated a family member’s cancer history without selecting “Self” were not considered cancer survivors. Individuals with skin cancer or multiple primary cancers were excluded. Individuals who indicated at least one family member, excluding themselves, were considered to have a positive family history of alcohol abuse [12]. Data for the current analysis were extracted from the All of Us Researcher Workbench and reflect cumulative survey responses collected since the program’s enrollment launch in 2018 through the date of extraction. Drinking behavior among cancer survivors was defined using three All of Us survey questions: (1) frequency of drinking (“How often did you have a drink containing alcohol in the past year?”; response options: never, monthly or less, 2–4 times a month, 2–3 times a week, ≥ 4 times a week); (2) quantity of drinking (“On a typical day when you drink, how many drinks do you have?”; response options: 1–2, 3–4, 5–6, 7–9, ≥ 10); and (3) binge drinking (“How often did you have 6 or more drinks on 1 occasion in the past year?”; response options: never, less than monthly, monthly, weekly, daily or almost daily). Moderate drinking was defined as reporting more than 2 drinks on a typical drinking day, and binge drinking was defined as ever consuming 6 or more drinks on a single occasion [12].

Cancers were categorized as alcohol-related (breast, colorectal, and head and neck) or non–alcohol-related [5]. Esophageal cancer was classified as non–alcohol-related because its association with alcohol consumption is largely confined to squamous cell carcinoma, whereas the majority of esophageal cancers in the USA are adenocarcinomas [29]. Liver cancer was not included among alcohol-related cancers because it was not specifically addressed in the survey items [12]. This study was conducted in accordance with the Declaration of Helsinki. All participants provided written informed consent to share EHRs, surveys, and other study data with qualified investigators for broad-based research. The Ohio State University IRB waived the requirement for further informed consent as the data were deidentified.

### Exposure and outcome of interest

The main exposure of interest was access to mental health care, which was assessed through the survey item: “In the past 12 months, was there any time when you needed any of the following, but didn’t get it because you couldn’t afford it: Mental health care or counseling?” Participants who responded “Yes” were classified as unable to access mental health care. Throughout this manuscript, “access” and “no access” are used as shorthand for this specific cost-related unmet need item, and do not imply the complete presence or absence of mental health treatment engagement.

### Assessment of other covariates

Information on age, sex, race and ethnicity, marital status, annual household income, and insurance status was obtained from the Basics survey, while general health condition was derived from the Overall Health survey. Marital status was categorized as cohabiting/married, divorced/separated, never married, widowed. Annual household income was categorized as > $150,000, $75,000–$150,000, $35,000–$75,000 and < $35,000. Low income was defined as an annual household income lower than $35,000. Data on preexisting mental illness were collected from the Personal and Family Health History survey and included self-reported diagnoses (“Including yourself, who in your family has had this condition?” with response “Self”) of the following conditions: depression anxiety reaction/panic disorder, social phobia, schizophrenia, bipolar disorder, attention-deficit/hyperactivity disorder (ADHD), autism, drug use disorder, eating disorder, personality disorder, post-traumatic stress disorder (PTSD), other mental health or substance use conditions, and alcohol abuse.

The Personal and Family Health History survey was used to ascertain family history of alcohol abuse (“Including yourself, who in your family has had alcohol use disorder?”). Participants who reported at least one family member, excluding themselves, were classified as having a positive family history of alcohol abuse. In addition, information on current treatment for both mental health conditions and cancer was obtained from the same survey (“Are you currently prescribed medications and/or receiving treatment for this condition?”; response options yes/no), as well as whether participants were still under medical care for cancer or mental health conditions (“Are you still seeing a doctor or health care provider for this condition?”).

### Statistical analysis

Descriptive statistics were reported as median values with interquartile range (IQR) for continuous variables and as frequency (%) for categorical variables. Comparisons between continuous and categorical variables were conducted using the Wilcoxon rank-sum test and the χ^2^ test or Fisher’s exact test, as appropriate. Univariable logistic regression was utilized to assess the association between covariates and risky drinking behaviors. Variables significant on univariable analyses (*p* < 0.05) were included in the multivariable models. Variables such as sex at birth, self-reported race, and ethnic background were included to reflect the demographic characteristics of the population (Table 1). Ethnicity was not included in the multivariable models given its substantial conceptual and empirical overlap with race, consistent with the approach used in other national health surveys; race was retained given its more granular categorization for the purposes of this analysis. Similarly, gender identity, rather than sex at birth, was used in the analytic models to reflect a more inclusive, self-identified demographic characteristic; the two variables showed nearly identical distributions within the cohort (Table 1), and their use was not expected to materially affect study findings. To incorporate the concept of intersectionality, logistic regression models were specified with higher-order interaction terms. Specifically, two-way, three-way, four-way, five-way, and six-way interactions among covariates were systematically evaluated, thereby capturing the joint effects of overlapping sociodemographic and Overall Health-related factors on the outcomes of interest [30–32].

**Table 1:** Baseline characteristics of overall cohort

Characteristic	*N*	Overall *N* = 18,839^*1*^	Access^3^ * N* = 17,827^*1*^	No access^3^ * N* = 1,012^*1*^	*p*-value^*2*^
**Gender identity**	18,839				** < 0.001**
Female		12,160.0 (64.5%)	11,330.0 (63.6%)	830.0 (82.0%)	
Male		6,476.0 (34.4%)	6,319.0 (35.4%)	157.0 (15.5%)	
Non-binary/transgender/others		203.0 (1.1%)	178.0 (1.0%)	25.0 (2.5%)	
**Sex at birth**	18,836				** < 0.001**
Female		12,190.0 (64.7%)	11,349.0 (63.7%)	841.0 (83.1%)	
Male		6,498.0 (34.5%)	6,338.0 (35.6%)	160.0 (15.8%)	
Not matching/others		148.0 (0.8%)	137.0 (0.8%)	11.0 (1.1%)	
**Race**	18,839				** < 0.001**
White		14,576.0 (77.4%)	13,892.0 (77.9%)	684.0 (67.6%)	
Asian		351.0 (1.9%)	328.0 (1.8%)	23.0 (2.3%)	
Black or African American		1,453.0 (7.7%)	1,353.0 (7.6%)	100.0 (9.9%)	
Underrepresented/others		2,459.0 (13.1%)	2,254.0 (12.6%)	205.0 (20.3%)	
**Ethnicity**	18,839				** < 0.001**
Hispanic or Latino		1,477.0 (7.8%)	1,351.0 (7.6%)	126.0 (12.5%)	
Not Hispanic or Latino		16,829.0 (89.3%)	15,975.0 (89.6%)	854.0 (84.4%)	
Others		533.0 (2.8%)	501.0 (2.8%)	32.0 (3.2%)	
**Self-reported race**	17,754				** < 0.001**
White		14,347.0 (80.8%)	13,689.0 (81.4%)	658.0 (70.5%)	
Asian		340.0 (1.9%)	318.0 (1.9%)	22.0 (2.4%)	
Black or African American		1,425.0 (8.0%)	1,329.0 (7.9%)	c	
Underrepresented/others		1,642.0 (9.2%)	1,485.0 (8.8%)	157.0 (16.8%)	
**Annual income**	16,637				** < 0.001**
> 150		3,392.0 (20.4%)	3,326.0 (21.1%)	66.0 (7.3%)	
75 k-150 k		5,293.0 (31.8%)	5,109.0 (32.5%)	184.0 (20.3%)	
35 k-75 k		4,356.0 (26.2%)	4,070.0 (25.9%)	286.0 (31.5%)	
< 35 k		3,596.0 (21.6%)	3,225.0 (20.5%)	371.0 (40.9%)	
**Health insurance**	18,658	18,391.0 (98.6%)	17,444.0 (98.8%)	947.0 (94.6%)	** < 0.001**
**Smoked ≥ 100 cigarettes (lifetime)**	18,520				0.4
No		10,759.0 (58.1%)	10,198.0 (58.2%)	561.0 (56.7%)	
Yes		7,761.0 (41.9%)	7,333.0 (41.8%)	428.0 (43.3%)	
**Marital status**	18,623				** < 0.001**
Cohabiting/married		11,899.0 (63.9%)	11,421.0 (64.8%)	478.0 (48.3%)	
Divorced/separated		3,193.0 (17.1%)	2,932.0 (16.6%)	261.0 (26.4%)	
Never married		2,124.0 (11.4%)	1,931.0 (11.0%)	193.0 (19.5%)	
Widowed		1,407.0 (7.6%)	1,350.0 (7.7%)	57.0 (5.8%)	
**Alcohol-related cancer**	18,839	6,774.0 (36.0%)	6,447.0 (36.2%)	327.0 (32.3%)	**0.013**
**Alcohol use frequency (past year)**	17,831				** < 0.001**
Never		3,411.0 (19.1%)	3,202.0 (19.0%)	209.0 (22.1%)	
Monthly or less		5,548.0 (31.1%)	5,173.0 (30.6%)	375.0 (39.6%)	
2–4 times/month		3,429.0 (19.2%)	3,260.0 (19.3%)	169.0 (17.8%)	
2–3 times/week		2,548.0 (14.3%)	2,448.0 (14.5%)	100.0 (10.6%)	
> 3 times/week		2,895.0 (16.2%)	2,801.0 (16.6%)	94.0 (9.9%)	
**Alcohol drinks per occasion**	14,184				** < 0.001**
1–2		12,250.0 (86.4%)	11,673.0 (86.8%)	577.0 (78.7%)	
3–4		1,550.0 (10.9%)	1,435.0 (10.7%)	115.0 (15.7%)	
5–6		279.0 (2.0%)	256.0 (1.9%)	23.0 (3.1%)	
7–9		61.0 (0.4%)	53.0 (0.4%)	8.0 (1.1%)	
> 9		44.0 (0.3%)	34.0 (0.3%)	10.0 (1.4%)	
**Binge drinking**	14,382				** < 0.001**
Never		10,875.0 (75.6%)	10,401.0 (76.2%)	474.0 (64.7%)	
Less than monthly		2,641.0 (18.4%)	2,447.0 (17.9%)	194.0 (26.5%)	
Monthly		546.0 (3.8%)	512.0 (3.8%)	34.0 (4.6%)	
Weekly		214.0 (1.5%)	193.0 (1.4%)	21.0 (2.9%)	
Daily or almost daily		106.0 (0.7%)	96.0 (0.7%)	10.0 (1.4%)	
**Family history of alcohol abuse**	18,831	247.0 (1.3%)	230.0 (1.3%)	17.0 (1.7%)	0.3
**Any mental health diagnosis**	18,839				** < 0.001**
No		11,492.0 (61.0%)	11,258.0 (63.2%)	234.0 (23.1%)	
Yes		7,347.0 (39.0%)	6,569.0 (36.8%)	778.0 (76.9%)	
**History of alcohol abuse**	18,831	746.0 (4.0%)	663.0 (3.7%)	83.0 (8.2%)	** < 0.001**
**Any mental health dx (among non alcohol abuser)**	18,831	6,599.0 (35.0%)	5,904.0 (33.1%)	695.0 (68.7%)	** < 0.001**
**Currently seeing provider for MH condition**	8,305	5,484.0 (66.0%)	4,905.0 (65.2%)	579.0 (73.6%)	** < 0.001**
**On medications/treatment for MH condition**	8,135	5,446.0 (66.9%)	4,875.0 (66.3%)	571.0 (73.3%)	** < 0.001**
**Currently seeing provider for cancer**	18,749	12,799.0 (68.3%)	12,149.0 (68.5%)	650.0 (64.4%)	**0.007**
**On medications/treatment for cancer**	18,723	7,394.0 (39.5%)	7,000.0 (39.5%)	394.0 (39.2%)	0.8

^*2*^Pearson's chi-squared test; Fisher’s exact test

^3^“Access” and “No access” refer to whether participants reported being able to afford needed mental health care or counseling in the past 12 months, and do not indicate the presence or absence of any mental health treatment engagement

For univariable logistic regression analyses, the Benjamini–Hochberg procedure was applied to control the False Discovery Rate (FDR) and adjust for multiple comparisons. Adjusted *q*-values were compared with raw *p*-values; because results were concordant (i.e., no discrepancies were observed between statistical significance of *p*- and *q*-values), FDR correction was not repeated in the multivariable models. Statistical significance was defined as a two-sided *p* < 0.05 [33–35]. All statistical analyses were performed using R version 4.2.2 within the *All of Us Researcher Workbench* (R Foundation for Statistical Computing, Vienna, Austria). Odds ratios (ORs) with 95% confidence intervals (CIs) were reported, and statistical significance was defined as *p* < 0.05.

## Results

### Baseline characteristics of overall cohort

The cohort included a total of 18,839 participants, the majority of whom were female (*n* = 12,160, 64.5%) and White (*n* = 14,576, 77.4%). Most participants reported an annual household income between $75,000 and $150,000 (*n* = 5293, 31.8%), followed by $35,000–75,000 (*n* = 4356, 26.2%), < $35,000 (*n* = 3596, 21.6%), and > $150,000 (*n* = 3392, 20.4%). Nearly all participants had health insurance coverage (*n* = 18,391, 98.6%). A total of 7761 individuals (41.9%) reported having smoked more than 100 cigarettes in their lifetime (Table 1). The most frequently reported marital status was cohabiting or married (*n* = 11,899, 63.9%), followed by divorced or separated (*n* = 3193, 17.1%), never married (*n* = 2124, 11.4%), and widowed (*n* = 1407, 7.6%). Among cancer diagnoses, 6774 (36.0%) were classified as alcohol-related cancers. Most participants (*n* = 12,799, 68.3%) reported currently seeing a doctor or health care provider for their cancer diagnosis, whereas only 39.5% (*n* = 7364) reported currently receiving medications or treatment for cancer.

Regarding alcohol consumption in the past year, the most frequent drinking pattern was monthly or less (*n* = 5548, 31.8%). Overall, 1934 participants (13.6%) reported exceeding moderate drinking, and 3507 (24.4%) reported binge drinking. A total of 7347 participants (39.0%) reported a lifetime history of at least one mental health condition. Among these individuals, 746 (4.0%) reported alcohol abuse, while 6599 (35.0%) reported a mental health condition other than alcohol abuse. Within this subgroup, most participants reported currently receiving medications or treatment for their mental health condition (*n* = 5446, 66.9%), as well as currently seeing a doctor or other health care provider for their mental health condition (*n* = 5484, 66.0%). Only 247 participants (1.3%) reported a family history of alcohol abuse (Table 1).

Among participants with at least one mental health diagnosis, the most frequently reported condition was major depression (*n* = 3999, 72.9%), followed by anxiety disorder (*n* = 2648, 48.2%) and post-traumatic stress disorder (PTSD; *n* = 1113, 20.3%) (Tables S1–S2). Comparing participants with and without binge drinking, individuals with binge drinking were more likely to have anxiety (52.2% vs 46.7%, *p* < 0.001), social phobia (5.0% vs 3.4%, *p* = 0.006), schizophrenia (1.5% vs 0.5%, *p* < 0.001), bipolar disorder (9.4% vs 6.2%, *p* < 0.001), ADHD (15.9% vs 12.7%, *p* < 0.001), drug abuse (6.0% vs 2.7%, *p* < 0.001), personality disorder (3.7% vs 2.1%, *p* < 0.001), and PTSD (22.7% vs 19.3%, *p* = 0.004). No difference was observed relative to depression. Similarly, compared with respondents who did not exceed moderate drinking, individuals who did report higher levels of drinking were more likely to report anxiety (51.6% vs 47.6%, *p* = 0.028), social phobia (6.3% vs 3.2%, *p* < 0.001), bipolar disorder (10.3% vs 6.5%, *p* < 0.001), ADHD (16.2% vs 13.0%, *p* = 0.009), personality disorder (4.5% vs 2.1%, *p* < 0.001), and PTSD (24.0% vs 19.6%, *p* = 0.003) (Table 1).

### Access to mental health care

A total of 1012 participants (5.4%) reported that in the past 12 months, they needed mental health counseling but were unable to obtain it due to cost. These individuals were twice as likely to have an annual income below $35,000 compared with individuals without this barrier (40.9% vs 20.5%, *p* < 0.001). Of note, these individuals were also more frequently divorced or separated (26.4% vs 16.6%) or never married (19.5% vs 11.0%). Participants who reported being unable to afford mental health counseling despite needing it had higher rates of binge drinking (35.3% vs 23.8%, *p* < 0.001) and exceeding moderate drinking (21.3% vs 13.2%, *p* < 0.001). Notably, most participants reported that they are currently seeing a doctor or other mental health care provider for their mental health condition (*n* = 579, 73.6%) and being under treatment or prescribed medications for their condition (*n* = 571, 73.3%) (Table 1). These data indicate that unmet mental health care need, as defined by this cost-related item, frequently coexisted with ongoing treatment engagement, rather than reflecting a complete absence of mental health care.

### Risk factors for risky drinking behaviors

On multivariable logistic regression, male sex was associated with 29% increased odds of binge drinking compared with females (OR 1.29, 95% CI 1.12–1.48, *p* < 0.001). Compared with White participants, Asian and Black individuals were not at higher risk of binge drinking (*p* = 0.12 and *p* = 0.2, respectively), whereas membership in other underrepresented groups was associated with 26% increased odds of excess drinking (OR 1.26, 95% CI 1.04–1.53, *p* = 0.018). Additional risk factors for binge drinking included low annual income (OR 1.28, 95% CI 1.09–1.50, *p* = 0.002) and having smoked more than 100 cigarettes in one’s lifetime (OR 1.53, 95% CI 1.35–1.74, *p* < 0.001). A history of mental illness (excluding alcohol abuse) was associated with higher odds of binge drinking (OR 1.70, 95% CI 1.37–2.12, *p* < 0.001), as was currently seeing a doctor or mental health care provider (OR 1.14, 95% CI 1.00–1.31, *p* = 0.048). Notably, reporting the need for but inability to afford mental health counseling in the past year was associated with 36% increased odds of binge drinking (OR 1.36, 95% CI 1.10–1.66, *p* = 0.004) (Table 2).

**Table 2:** Univariable an multivariable logistic regression for binge drinking

Variable	Univariable			Multivariable	
	OR (95% CI)	*p*-value	*q*-value^*1*^	OR (95% CI)	*p*-value
**Gender**					
Female					
Male	1.4(1.30, 1.51)	** < 0.001**	** < 0.001**	1.29(1.12, 1.48)	** < 0.001**
Non-binary/transgender/others	1.51(1.05, 2.14)	**0.021**	**0.03**	1.17(0.68, 1.96)	0.6
**Race**					
White					
Asian	1.13(0.82, 1.52)	0.4	0.5	1.65(0.85, 3.05)	0.12
Black or African American	1.66(1.44, 1.91)	** < 0.001**	** < 0.001**	1.18(0.90, 1.54)	0.2
Underrepresented/others	1.42(1.26, 1.59)	** < 0.001**	** < 0.001**	1.26(1.04, 1.53)	**0.018**
**Low income**					
No					
Yes	1.62(1.47, 1.79)	** < 0.001**	** < 0.001**	1.28(1.09, 1.50)	**0.002**
**Health insurance**					
Yes					
No	2.03(1.50, 2.72)	** < 0.001**	** < 0.001**	1.23(0.74, 2.00)	0.4
**Smoked > 100 cigarettes**					
No					
Yes	1.59(1.47, 1.71)	** < 0.001**	** < 0.001**	1.53(1.35, 1.74)	** < 0.001**
**Marital status**					
Cohabiting/married					
Divorced/separated	1.16(1.04, 1.28)	**0.006**	**0.009**	0.83(0.69, 0.98)	**0.029**
Never married	1.55(1.38, 1.74)	** < 0.001**	** < 0.001**	1.07(0.87, 1.30)	0.5
Widowed	0.8(0.68, 0.93)	**0.006**	**0.009**	0.67(0.50, 0.89)	**0.006**
**Family history of alcohol abuse**					
No					
Yes	1.02(0.71, 1.43)	> 0.9	> 0.9		
**History of alcohol abuse**					
No					
Yes	7.12(5.68, 8.97)	** < 0.001**	** < 0.001**	8.12(5.92, 11.2)	** < 0.001**
**Other mental health diagnosis**					
No					
Yes	1.14(1.05, 1.23)	**0.001**	**0.002**	1.7(1.37, 2.12)	** < 0.001**
**Still seeing a doctor for mental health condition**					
No					
Yes	1.14(1.01, 1.28)	**0.028**	**0.038**	1.14(1.00, 1.31)	**0.048**
**Stil under treatment for mental health condition**					
No					
Yes	1.11(0.98, 1.25)	0.09	0.12		
**Still seeing a doctor for cancer**					
No					
Yes	1.05(0.97, 1.14)	0.3	0.3		
**Still under treatment for cancer**					
No					
Yes	1.06(0.99, 1.15)	0.11	0.14		
**Mental health care**					
Access					
No access	1.75(1.49, 2.04)	** < 0.001**	** < 0.001**	1.36(1.10, 1.66)	**0.004**

^*1*^False discovery rate correction for multiple testing

Abbreviations: *CI*, confidence interval; *OR*, odds ratio

Among individuals who reported more than moderate drinking, factors independently associated with excess drinking included male sex (OR 1.67, 95% CI 1.49–1.86, *p* < 0.001), low annual income (OR 1.31, 95% CI 1.13–1.52, *p* < 0.001), lack of health insurance (OR 1.9, 95% CI 1.27–2.79, *p* = 0.001), smoking more than 100 cigarettes in a lifetime (OR 2.12, 95% CI 1.91–2.79, *p* = 0.001), and never having been married (OR 1.43, 95% CI 1.20–1.70, *p* < 0.001). Interestingly, a history of mental illness (excluding alcohol abuse) was not associated with exceeding moderate drinking (*p* = 0.5). In contrast, being unable to afford mental health counseling when needed in the past year was associated with 63% increased odds of excess drinking, even after adjusting for a previous history of alcohol abuse (OR 1.63, 95% CI 1.31–2.01, *p* < 0.001) (Table 3). No interactions among social determinants of overall health with binge drinking or exceeding moderate drinking were noted (all *p* > 0.05). Notably, the univariable association between Black race and both binge drinking and exceeding moderate drinking was attenuated and no longer statistically significant after multivariable adjustment. This finding suggests that this association may be substantially explained by other socioeconomic and structural covariates included in the model, such as income, insurance status, and marital status.

**Table 3:** Univariable an multivariable logistic regression for Exceeding moderate drinking

Variable	Univariable			Multivariable	
	OR(95% CI)	*p*-value	*q*-value^*1*^	OR(95% CI)	*p*-value
**Gender**					
Female					
Male	1.7(1.55, 1.87)	** < 0.001**	** < 0.001**	1.67(1.49, 1.86)	** < 0.001**
Non-binary/transgender/others	1.79(1.15, 2.69)	**0.007**	**0.012**	1.31(0.76, 2.15)	0.3
**Race**					
White					
Asian	0.74(0.45, 1.13)	0.2	0.2	0.73(0.42, 1.19)	0.2
Black or African American	1.4(1.17, 1.67)	** < 0.001**	** < 0.001**	1.08(0.87, 1.34)	0.5
Underrepresented/others	1.36(1.18, 1.57)	** < 0.001**	** < 0.001**	1.22(1.03, 1.43)	**0.022**
**Low income**					
No					
Yes	1.81(1.61, 2.03)	** < 0.001**	** < 0.001**	1.31(1.13, 1.52)	** < 0.001**
**Health insurance**					
Yes					
No	2.61(1.87, 3.59)	** < 0.001**	** < 0.001**	1.9(1.27, 2.79)	**0.001**
**Smoked > 100 cigarettes**					
No					
Yes	2.37(2.15, 2.61)	** < 0.001**	** < 0.001**	2.12(1.91, 2.37)	** < 0.001**
**Marital status**					
Cohabiting/married					
Divorced/separated	1.35(1.19, 1.53)	** < 0.001**	** < 0.001**	1.12(0.96, 1.31)	0.14
Never married	1.63(1.41, 1.88)	** < 0.001**	** < 0.001**	1.43(1.20, 1.70)	** < 0.001**
Widowed	0.82(0.66, 1.01)	0.071	0.1	0.76(0.60, 0.97)	**0.031**
**Family history of alcohol abuse**					
No					
Yes	1.47(0.97, 2.14)	0.056	0.08		
**History of alcohol abuse**					
No					
Yes	10.6(8.50, 13.2)	** < 0.001**	** < 0.001**	7.24(5.68, 9.26)	** < 0.001**
**Other mental health diagnosis**					
No					
Yes	1.03(0.94, 1.14)	0.5	0.5		
**Still seeing a doctor for mental health condition**					
No					
Yes	1.03(0.89, 1.19)	0.7	0.7		
**Stil under treatment for mental health condition**					
No					
Yes	1.04(0.90, 1.20)	0.6	0.6		
**Still seeing a doctor for cancer**					
No					
Yes	1.04(0.94, 1.15)	0.4	0.5		
**Still under treatment for cancer**					
No					
Yes	1.03(0.94, 1.14)	0.5	0.5		
**Mental health care**					
Access					
No access	1.78(1.47, 2.13)	** < 0.001**	** < 0.001**	1.63(1.31, 2.01)	** < 0.001**

^*1*^False discovery rate correction for multiple testing

Abbreviations: *CI*, confidence interval; *OR*, odds ratio

### Sub-analysis on patients without history of mental health conditions

A sub-analysis was conducted on 11,492 participants without a history of alcohol abuse or other mental health conditions. In this sub-cohort, participants who reported being unable to afford mental health counseling in the past 12 months were more likely to have a low annual income (30.1% vs 15.7%, *p* < 0.001), and no health insurance coverage (94.1% vs 98.9%, *p* < 0.001). Participants unable to access mental health care reported higher rates of excess moderate drinking (19.0% vs 11.6%, *p* = 0.038) and binge drinking (36.5% vs 21.6%, *p* < 0.001). No differences were noted in drinking frequency over the past year, family history of alcohol abuse, or being currently in treatment or under the care of a doctor/health care provider for cancer (Table S3).

Within this sub-cohort of 11,492 respondents, on multivariable analysis, factors associated with excess drinking included male sex (OR 1.84, 95% CI 1.59–2.12, *p* < 0.001), membership in underrepresented racial/ethnic groups (OR 1.35, 95% CI 1.07–1.68, *p* = 0.009), and smoking more than 100 cigarettes in a lifetime (OR 2.17, 95% CI 1.88–2.51, *p* < 0.001). In addition, lack of health insurance was associated with 86% increased odds of excessive drinking (OR 1.86, 95% CI 1.03–3.22, *p* = 0.033), while inability to afford mental health care was associated with 77% increased odds (OR 1.77, 95% CI 1.10–2.75, *p* = 0.014) (Tables S4 and S5).

## Discussion

The interplay of a cancer diagnosis, mental health needs, and risky drinking behaviors is complex. Cancer patients often experience increased anxiety, depression, and other psychological challenges related to their malignant diagnosis [36]. Among cancer survivors, anxiety is a common experience related to fears of cancer recurrence, the uncertainty of follow-up care, physical changes from treatment, and difficulties returning to daily life [37]. Drinking alcohol is a coping mechanism that can be adopted by a subset of individuals. Use of alcohol as a coping motive has been associated with particularly poor outcomes, including worsening of mental health conditions and overall quality of life [38]. While the reasons to “self-medicate” with alcohol are multifaceted, the inability to access mental health services may contribute to patient use of substances. To this point, Harris et al. reported that substance use varied with past year unmet need for mental health care and mental health care use in ways consistent with the self-medication hypothesis [39]. While this study focused on the general US population, the authors suggested that future research was needed to investigate sub-groups of individuals who were at particular risk to target interventions. As such, the current study was important as we specifically characterized the impact of mental health care access on drinking behaviors among cancer survivors. Among 18,839 cancer survivors included in the analytic cohort, 1 in 20 reported an inability to afford mental health counseling despite a perceived need. Importantly, among individuals who reported an unmet need for mental health services, the prevalence of excessive drinking (21.3% vs 13.2%, *p* < 0.001) and binge drinking (35.3% vs 23.8%, *p*< 0.001) was markedly higher. In fact, on multivariable analysis after controlling for other measured confounders, inability to access mental health care was independently associated with 48% and 36% higher odds of excessive and binge drinking, respectively, even among participants without a preexisting mental illness. Prior studies have separately characterized the prevalence of unmet mental health needs among cancer survivors and the association between unmet mental health need and substance use in the general population [12, 40, 41]. However, to our knowledge no study has directly examined this relationship within a cancer survivor cohort. These findings underscore the importance of expanding screening and access to mental health care to maximize treatment opportunities and improve the quality of life of cancer survivors, regardless of whether they have a history of mental health conditions.

With improvements in the quality of oncologic care, patients are living longer, and the number of cancer survivors has markedly increased in the USA over the last two decades [42]. Cancer survivors often face unique social and psychological stressors, including anxiety, depression, cognitive effects of chemotherapy, as well as fear of cancer recurrence [36]. In fact, the prevalence of depression and anxiety among cancer patients has been reported to be 20% and 10%, respectively, while 30–35% of patients have a more severe concomitant psychiatric disorder [42, 43]. Paredes et al. reported that patients with early-stage pancreatic cancer were less likely to undergo treatment if they had a history of mental illness and had worse long-term cancer-related outcomes, even after adjusting for disease-specific factors [44]. Other reports have noted that individuals with cancer and comorbid mental health issues have a life expectancy 3–5 years lower than other surviors [45, 46]. In the current study, 39% of patients reported a history of mental illness with the most frequently reported conditions being depression, anxiety, and PTSD. Importantly, among patients with mental health illness only two-thirds reported receiving treatment. Somewhat surprisingly, 1 in 20 individuals reported that in the past 12 months a need for mental health counseling but an inability to obtain it due to cost. Coombs et al. had similarly noted that the most prevalent barriers to mental health care access were linked to issues with affordability, and mental health challenges existed more often when any barrier was reported [47]. In our cohort, individuals needing mental health counseling but unable to obtain it due to cost were twice as likely to have an annual income below $35,000. Collectively, these data highlight the need to make mental health resources more affordable and accessible to cancer survivors. Interestingly, while Black patients had increased odds of both binge drinking and exceeding moderate drinking on univariable analysis, this association was attenuated after adjustment. This pattern suggests that racial disparities in risky drinking behavior observed in our cohort may be substantially mediated by socioeconomic and structural inequities rather than representing an independent effect of race itself. This finding is consistent with prior work demonstrating that racial disparities in alcohol-related outcomes are often attenuated after accounting for socioeconomic status and health care access [48].

Shi et al. reported that 77.7% of cancer survivors were current drinkers; 13% of patients exceeded moderate drinking and 23.8% reported binge drinking [12]. Similarly, in the current study, 13.6% of patients reported exceeding moderate drinking and 24.4% reported binge drinking. While the reason is undoubtedly multifactorial, alcohol consumption among cancer survivors has been linked to mental health. Specifically, patients with mental illness often have an increased risk of developing alcohol use disorder, which is consistent across different types of psychiatric diagnoses [49]. For example, among individuals with anxiety or phobic disorders, the prevalence of alcohol use disorder was 17%, while among cancer survivors with mood disorders the prevalence was 11%—roughly twofold higher than individuals without a mental health diagnosis. Among cancer patients receiving psycho-oncology support 15% of individuals engaged in risky alcohol behaviors with binge drinking occurred in 4.4% and 8.7% of men and women, respectively [50]. In the current study, while not associated with excess moderate drinking, a history of mental health illness was independently associated with 70% increased odds of binge drinking. Of particular note, being unable to afford mental health care was associated with increased odds of excess moderate and/or binge drinking, even after adjusting for a previous history of alcohol abuse. In addition, even on subset analysis that included only patients without a history of alcohol abuse and a mental health diagnosis, inability to afford mental health care was associated with 77% increased odds of excess drinking. In turn, excessive drinking can result in increased risk of recurrence and development of second primary cancers with subsequent elevated mortality [8, 9, 51–57]. Moreover, alcohol consumption adversely affects postoperative outcomes, being associated with higher complication rates, longer hospital stays, greater need for surgical procedures, and ultimately increased health care costs [58–60].

Supportive care plays a central role in survivorship, especially in addressing mental health needs of cancer patients and improving their quality of life [61]. Possible interventions include counseling, psychotherapy, and medication management, which may help patients cope with the burden of cancer and improve the long-term effects of treatment [62–64]. Counseling and psychotherapy can assist patients to develop coping strategies through relaxation techniques, mindfulness, and cognitive-behavioral therapy [65, 66].Accordingly, access to mental health care becomes a crucial determinant of both oncologic outcomes and quality of life. Unfortunately, approximately 15% of privately insured patients and 11% of Medicare beneficiaries experienced difficulties scheduling appointments with mental health counselors [67]. In the current study, 5% of respondents reported needing but being unable to access mental health counseling in the past 12 months due to cost. Of note, among participants with a preexisting mental illness, 73.6% of individuals who were unable to access counseling despite reporting a need were still seeing a health care provider. As such, integrating mental health services into the primary care of cancer survivors may be an important means to improve access [68].

Results of the current study should be interpreted in light of several limitations. The Dietary Guidelines for Americans 2020–2025 define exceeding moderate drinking for women as consuming more than one drink on a drinking day, and the National Institute on Alcohol Abuse and Alcoholism defines binge drinking as ≥ 4 drinks on a single occasion for women and ≥ 5 for men. In contrast, the All of Us survey defined these thresholds as > 2 drinks on a typical drinking day and ≥ 6 drinks on a single occasion. Consequently, the definitions used in the current analysis may have underestimated the true prevalence of risky drinking behaviors [12]. In addition, given the cross-sectional nature of survey data, the exact timing of alcohol consumption, cancer diagnosis and treatment, and mental health diagnoses could not be established, although alcohol consumption behaviors and mental health care access were explicitly framed within the previous 12 months. Additionally, while cancer diagnoses were categorized descriptively as alcohol-related versus non–alcohol-related (Table 1), cancer type was not evaluated as a candidate predictor in the univariable or multivariable regression models for drinking behavior, as the primary analytic focus of this study was mental health care access rather than cancer-specific characteristics. A full stratification of risk factors by individual cancer site was similarly beyond the scope of the current study, given the heterogeneity of cancer diagnoses represented in this cohort and the need for adequately powered subgroups to support such an analysis. Another inherent limitation of survey-based studies was that not all participants answered every question analyzed in this study, leading to some missing data across different reported answers. Missing data were not imputed, and all analyses were conducted using available cases; this approach assumes data were missing completely at random, which could not be formally verified and may have introduced bias if missingness was related to unmeasured factors. Our exposure variable was limited to a single item assessing cost-related barriers to mental health care, as this was the only survey item in the All of Us dataset that isolated mental health care as a distinct category of unmet need. While the All of Us Health Care Access and Utilization survey includes additional items on other barriers to care (e.g., transportation, distance to provider, inability to take time off work), these items pertain to delayed medical care generally and could not be attributed specifically to mental health care. As such, our definition of “no access to mental health care” reflects affordability barriers only and does not capture other potentially important dimensions of access such as provider availability, geographic proximity, or stigma.

In conclusion, prompt and equitable access to mental health care remains an unmet need among cancer survivors, both among individuals with and without a history of mental illness. Up to 1 in 20 cancer survivors self-reported a need for mental health services within the last year yet were unable to attain such care due to cost. Limited access to mental health services was associated with an increased risk of risky drinking behaviors and excessive alcohol consumption. Ensuring and expanding access to mental health services and effectively integrating them into the continuum of oncologic care should be a priority within cancer survivorship programs.

## Supplementary Information

Below is the link to the electronic supplementary material.

ESM 1 (DOCX 37.8 KB)

## Data Availability

The data that support the findings of the study are available from All of Us Research Program. Restrictions apply to the availability of these data, which were used under license for this study. Data are available from http://allofus.nih.gov/ with the permission of All of Us Research Program.
