# Supplementary material for: Inaccessible mental health care is a risk factor for risky drinking behaviors among cancer survivors: an All of Us survey-based cohort study
Source: Support Care Cancer. 2026 Sep 17;34(10):983. doi: 10.1007/s00520-026-11235-3 (PMC13586038; doi:10.1007/s00520-026-11235-3)
Supplement: Supplementary file 1 [file 520_2026_11235_MOESM1_ESM.docx]

**Table S1**. Mental health diagnosis, excluding alcohol disorders, according to Binge drinking.

| **Characteristic** | **N** | **Overall** N = 14,673*^1^* | **No Binge Drinking** N = 11,096*^1^* | **Binge Drinking** N = 3,577*^1^* | **p-value***^2^* |
| --- | --- | --- | --- | --- | --- |
| **Anxiety** | 5,489 | 2,648.0 (48.2%) | 1,827.0 (46.7%) | 821.0 (52.2%) | **<0.001** |
| **Autism** | 5,489 | 55.0 (1.0%) | 35.0 (0.9%) | 20.0 (1.3%) | 0.2 |
| **Depression** | 5,489 | 3,999.0 (72.9%) | 2,870.0 (73.3%) | 1,129.0 (71.7%) | 0.2 |
| **Social Phobia** | 5,489 | 210.0 (3.8%) | 132.0 (3.4%) | 78.0 (5.0%) | **0.006** |
| **Schizophrenia** | 5,489 | 44.0 (0.8%) | 21.0 (0.5%) | 23.0 (1.5%) | **<0.001** |
| **Bipolar** | 5,489 | 391.0 (7.1%) | 243.0 (6.2%) | 148.0 (9.4%) | **<0.001** |
| **Other** | 5,489 | 153.0 (2.8%) | 107.0 (2.7%) | 46.0 (2.9%) | 0.7 |
| **ADHD** | 5,489 | 747.0 (13.6%) | 496.0 (12.7%) | 251.0 (15.9%) | **0.001** |
| **Drug abuse** | 5,489 | 199.0 (3.6%) | 104.0 (2.7%) | 95.0 (6.0%) | **<0.001** |
| **Eating disorder** | 5,489 | 383.0 (7.0%) | 267.0 (6.8%) | 116.0 (7.4%) | 0.5 |
| **Personality disorder** | 5,489 | 140.0 (2.6%) | 82.0 (2.1%) | 58.0 (3.7%) | **<0.001** |
| **PTSD** | 5,489 | 1,113.0 (20.3%) | 755.0 (19.3%) | 358.0 (22.7%) | **0.004** |
| *^1^*n (%) | | | | | |
| *^2^*Pearson's Chi-squared test | | | | | |

**Table S2.** Mental health diagnosis, excluding alcohol disorders, according to exceeding moderate drinking.

| **Characteristic** | **N** | **Overall** N = 14,471*^1^* | **Not exceeded** N = 12,504*^1^* | **Exceeded** N = 1,967*^1^* | **p-value***^2^* |
| --- | --- | --- | --- | --- | --- |
| **Anxiety** | 5,416 | 2,614.0 (48.3%) | 2,144.0 (47.6%) | 470.0 (51.6%) | **0.028** |
| **Autism** | 5,416 | 55.0 (1.0%) | 42.0 (0.9%) | 13.0 (1.4%) | 0.2 |
| **Depression** | 5,416 | 3,950.0 (72.9%) | **3,308.0 (73.4%)** | 642.0 (70.5%) | 0.067 |
| **Social Phobia** | 5,416 | 202.0 (3.7%) | 145.0 (3.2%) | 57.0 (6.3%) | **<0.001** |
| **Schizophrenia** | 5,416 | 43.0 (0.8%) | 31.0 (0.7%) | 12.0 (1.3%) | 0.051 |
| **Bipolar** | 5,416 | 388.0 (7.2%) | 294.0 (6.5%) | 94.0 (10.3%) | **<0.001** |
| **Other** | 5,416 | 149.0 (2.8%) | 115.0 (2.6%) | 34.0 (3.7%) | **0.047** |
| **ADHD** | 5,416 | 734.0 (13.6%) | 586.0 (13.0%) | 148.0 (16.2%) | **0.009** |
| **Drug abuse** | 5,416 | 195.0 (3.6%) | 116.0 (2.6%) | 79.0 (8.7%) | **<0.001** |
| **Eating disorder** | 5,416 | 379.0 (7.0%) | 313.0 (6.9%) | 66.0 (7.2%) | 0.7 |
| **Personality disorder** | 5,416 | 135.0 (2.5%) | 94.0 (2.1%) | 41.0 (4.5%) | **<0.001** |
| **PTSD** | 5,416 | 1,103.0 (20.4%) | 884.0 (19.6%) | 219.0 (24.0%) | **0.003** |
| *^1^*n (%) | | | | | |
| *^2^*Pearson's Chi-squared test | | | | | |

**Table S3.** Baseline characteristics of patients with no history of mental health diagnosis including alcohol abuse.

| **Characteristic** | **N** | **Overall** N = 11,492*^1^* | **no** N = 11,258*^1^* | **yes** N = 234*^1^* | **p-value***^2^* |
| --- | --- | --- | --- | --- | --- |
| **Gender identity** | 11,492 |  |  |  | **<0.001** |
| Female |  | 6,841.0 (59.5%) | 6,658.0 (59.1%) | 183.0 (78.2%) |  |
| Male |  | 4,540.0 (39.5%) | 4,492.0 (39.9%) | 48.0 (20.5%) |  |
| Non Binary/Transgender/others |  | 111.0 (1.0%) | 108.0 (1.0%) | 3.0 (1.3%) |  |
| **Sex at birth** | 11,491 |  |  |  | **<0.001** |
| Female |  | 6,848.0 (59.6%) | 6,664.0 (59.2%) | 184.0 (78.6%) |  |
| Male |  | 4,549.0 (39.6%) | 4,501.0 (40.0%) | 48.0 (20.5%) |  |
| Not matching/others |  | 94.0 (0.8%) | 92.0 (0.8%) | 2.0 (0.9%) |  |
| **Race** | 11,492 |  |  |  | **<0.001** |
| White |  | 8,805.0 (76.6%) | 8,658.0 (76.9%) | 147.0 (62.8%) |  |
| Asian |  | 274.0 (2.4%) | 263.0 (2.3%) | 11.0 (4.7%) |  |
| Black or African American |  | 959.0 (8.3%) | 931.0 (8.3%) | 28.0 (12.0%) |  |
| Underrepresented/ others |  | 1,454.0 (12.7%) | 1,406.0 (12.5%) | 48.0 (20.5%) |  |
| **Ethnicity** | 11,492 |  |  |  | **<0.001** |
| Hispanic or Latino |  | 915.0 (8.0%) | 874.0 (7.8%) | 41.0 (17.5%) |  |
| Not Hispanic or Latino |  | 10,252.0 (89.2%) | 10,065.0 (89.4%) | 187.0 (79.9%) |  |
| Others |  | 325.0 (2.8%) | 319.0 (2.8%) | 6.0 (2.6%) |  |
| **Self-reported race** | 10,781 |  |  |  | **<0.001** |
| White |  | 8,679.0 (80.5%) | 8,540.0 (80.8%) | 139.0 (67.5%) |  |
| Asian |  | 268.0 (2.5%) | 257.0 (2.4%) | 11.0 (5.3%) |  |
| Black or African American |  | 945.0 (8.8%) | 919.0 (8.7%) | 26.0 (12.6%) |  |
| Underrepresented/ others |  | 889.0 (8.2%) | 859.0 (8.1%) | 30.0 (14.6%) |  |
| **Annual income** | 10,002 |  |  |  | **<0.001** |
| >150 |  | 2,410.0 (24.1%) | 2,383.0 (24.3%) | 27.0 (13.8%) |  |
| 75k-150k |  | 3,390.0 (33.9%) | 3,333.0 (34.0%) | 57.0 (29.1%) |  |
| 35k-75k |  | 2,607.0 (26.1%) | 2,554.0 (26.0%) | 53.0 (27.0%) |  |
| < 35k |  | 1,595.0 (15.9%) | 1,536.0 (15.7%) | 59.0 (30.1%) |  |
| **Health insurance** | 11,379 | 11,247.0 (98.8%) | 11,027.0 (98.9%) | 220.0 (94.8%) | **<0.001** |
| **Smoked ≥100 cigarettes (lifetime)** | 11,304 |  |  |  | 0.085 |
| 0 |  | 7,031.0 (62.2%) | 6,878.0 (62.1%) | 153.0 (67.7%) |  |
| 1 |  | 4,273.0 (37.8%) | 4,200.0 (37.9%) | 73.0 (32.3%) |  |
| **Marital status** | 11,369 |  |  |  | **<0.001** |
| Cohabiting/Married |  | 7,795.0 (68.6%) | 7,674.0 (68.9%) | 121.0 (53.1%) |  |
| Divorced/Separated |  | 1,560.0 (13.7%) | 1,508.0 (13.5%) | 52.0 (22.8%) |  |
| Never married |  | 1,105.0 (9.7%) | 1,064.0 (9.6%) | 41.0 (18.0%) |  |
| Widowed |  | 909.0 (8.0%) | 895.0 (8.0%) | 14.0 (6.1%) |  |
| **Alcohol-related cancer** | 11,492 | 4,171.0 (36.3%) | 4,073.0 (36.2%) | 98.0 (41.9%) | 0.073 |
| **Alcohol use frequency (past year)** | 10,810 |  |  |  | 0.069 |
| Never |  | 1,782.0 (16.5%) | 1,742.0 (16.4%) | 40.0 (19.1%) |  |
| Monthly or less |  | 3,187.0 (29.5%) | 3,113.0 (29.4%) | 74.0 (35.4%) |  |
| 2-4 times/month |  | 2,221.0 (20.5%) | 2,180.0 (20.6%) | 41.0 (19.6%) |  |
| 2-3 times/week |  | 1,678.0 (15.5%) | 1,648.0 (15.5%) | 30.0 (14.4%) |  |
| >3 times/week |  | 1,942.0 (18.0%) | 1,918.0 (18.1%) | 24.0 (11.5%) |  |
| **Alcohol drinks per occasion** | 8,880 |  |  |  | **0.038** |
| 1-2 |  | 7,840.0 (88.3%) | 7,704.0 (88.4%) | 136.0 (81.0%) |  |
| 3-4 |  | 883.0 (9.9%) | 854.0 (9.8%) | 29.0 (17.3%) |  |
| 5-6 |  | 119.0 (1.3%) | 116.0 (1.3%) | 3.0 (1.8%) |  |
| 7-9 |  | 23.0 (0.3%) | 23.0 (0.3%) | 0.0 (0.0%) |  |
| >9 |  | 15.0 (0.2%) | 15.0 (0.2%) | 0.0 (0.0%) |  |
| **Binge drinking** | 9,006 |  |  |  | **<0.001** |
| Never |  | 7,038.0 (78.1%) | 6,932.0 (78.4%) | 106.0 (63.5%) |  |
| Less than monthly |  | 1,510.0 (16.8%) | 1,463.0 (16.6%) | 47.0 (28.1%) |  |
| Monthly |  | 314.0 (3.5%) | 303.0 (3.4%) | 11.0 (6.6%) |  |
| Weekly |  | 101.0 (1.1%) | 99.0 (1.1%) | 2.0 (1.2%) |  |
| Daily or almost daily |  | 43.0 (0.5%) | 42.0 (0.5%) | 1.0 (0.6%) |  |
| **Family history of alcohol abuse** | 11,486 | 102.0 (0.9%) | 102.0 (0.9%) | 0.0 (0.0%) | 0.3 |
| **Currently seeing provider for cancer** | 11,433 | 7,863.0 (68.8%) | 7,696.0 (68.7%) | 167.0 (71.7%) | 0.3 |
| **On medications/treatment for cancer** | 11,412 | 4,373.0 (38.3%) | 4,272.0 (38.2%) | 101.0 (43.5%) | 0.1 |
| ***^1^*n (%)** | | | | | |
| ***^2^*Fisher's exact test; Pearson's Chi-squared test** | | | | | |

**Table S4.** Univariable an multivariable logistic regression for binge drinking among patients with no history of mental illness including alcohol abuse.

|  | **Univariable** | | **Multivariable** | |
| --- | --- | --- | --- | --- |
| **Variable** | **OR** | **p-value** |  |  |
| **Gender** |  |  |  |  |
| Female |  |  |  |  |
| Male | 1.49(1.35, 1.65) | **<0.001** | 1.5(1.34, 1.67) | **<0.001** |
| Non-Binary/Transgender/others | 1.61(0.95, 2.62) | 0.067 | 1.32(0.69, 2.37) | 0.4 |
| **Race** |  |  |  |  |
| White |  |  |  |  |
| Asian | 1.06(0.72, 1.52) | 0.8 | 1.22(0.82, 1.79) | 0.3 |
| Black or African American | 1.79(1.50, 2.14) | **<0.001** | 1.41(1.14, 1.75) | **0.001** |
| Underrepresented/ others | 1.49(1.28, 1.73) | **<0.001** | 1.32(1.10, 1.57) | **0.002** |
| **Low income** |  |  |  |  |
| No |  |  |  |  |
| Yes | 1.59(1.37, 1.84) | **<0.001** | 1.35(1.14, 1.60) | **<0.001** |
| **Health insurance** |  |  |  |  |
| Yes |  |  |  |  |
| No | 2.07(1.33, 3.17) | **<0.001** | 1.52(0.90, 2.51) | 0.11 |
| **Smoked >100 cigarettes** |  |  |  |  |
| No |  |  |  |  |
| Yes | 1.46(1.32, 1.61) | **<0.001** | 1.41(1.27, 1.58) | **<0.001** |
| **Marital Status** |  |  |  |  |
| Cohabiting/Married |  |  |  |  |
| Divorced/Separated | 1.18(1.02, 1.37) | **0.027** | 1(0.84, 1.19) | >0.9 |
| Never married | 1.69(1.44, 1.99) | **<0.001** | 1.48(1.23, 1.79) | **<0.001** |
| Widowed | 0.8(0.65, 0.98) | **0.037** | 0.74(0.58, 0.94) | **0.014** |
| **Family history of alcohol abuse** |  |  |  |  |
| No |  |  |  |  |
| Yes | 0.82(0.44, 1.43) | 0.5 |  |  |
| **Still seeing a doctor for cancer** |  |  |  |  |
| No |  |  |  |  |
| Yes | 1.07(0.96, 1.19) | 0.2 |  |  |
| **Still under treatment for cancer** |  |  |  |  |
| No |  |  |  |  |
| Yes | 1.08(0.98, 1.20) | 0.14 |  |  |
| **Mental health care** |  |  |  |  |
| Access |  |  |  |  |
| No access | 2.09(1.51, 2.87) | **<0.001** | 1.88(1.30, 2.70) | **<0.001** |
| Abbreviations: CI = Confidence Interval, OR = Odds Ratio | | |  |  |

**Table S5.** Univariable an multivariable logistic regression for exceeding moderate drinking among patients with no history of mental illness including alcohol abuse.

|  | **Univariable** | | **Multivariable** | |
| --- | --- | --- | --- | --- |
| **Variable** | **95% CI** | **p-value** |  |  |
| **Gender** |  |  |  |  |
| Female |  |  |  |  |
| Male | 1.86(1.64, 2.12) | **<0.001** | 1.84(1.59, 2.12) | **<0.001** |
| Non-Binary/Transgender/others | 2.43(1.33, 4.19) | **0.002** | 1.88(0.85, 3.72) | 0.091 |
| **Race** |  |  |  |  |
| White |  |  |  |  |
| Asian | 0.52(0.26, 0.94) | **0.047** | 0.48(0.21, 0.93) | **0.048** |
| Black or African American | 1.59(1.27, 1.99) | **<0.001** | 1.43(1.09, 1.85) | **0.009** |
| Underrepresented/ others | 1.43(1.18, 1.73) | **<0.001** | 1.35(1.07, 1.68) | **0.009** |
| **Low income** |  |  |  |  |
| No |  |  |  |  |
| Yes | 1.61(1.34, 1.93) | **<0.001** | 1.29(1.04, 1.60) | **0.02** |
| **Health insurance** |  |  |  |  |
| Yes |  |  |  |  |
| No | 2.49(1.50, 3.97) | **<0.001** | 1.86(1.03, 3.22) | **0.033** |
| **Smoked >100 cigarettes** |  |  |  |  |
| No |  |  |  |  |
| Yes | 2.29(2.01, 2.62) | **<0.001** | 2.17(1.88, 2.51) | **<0.001** |
| **Marital Status** |  |  |  |  |
| Cohabiting/Married |  |  |  |  |
| Divorced/Separated | 1.19(0.98, 1.44) | 0.067 | 1.1(0.87, 1.37) | 0.4 |
| Never married | 1.62(1.32, 1.97) | **<0.001** | 1.49(1.17, 1.88) | **0.001** |
| Widowed | 0.91(0.69, 1.17) | 0.5 | 0.85(0.62, 1.15) | 0.3 |
| **Family history of alcohol abuse** |  |  |  |  |
| No |  |  |  |  |
| Yes | 1.22(0.59, 2.29) | 0.6 |  |  |
| **Still seeing a doctor for cancer** |  |  |  |  |
| No |  |  |  |  |
| Yes | 1.08(0.94, 1.24) | 0.3 |  |  |
| **Still under treatment for cancer** |  |  |  |  |
| No |  |  |  |  |
| Yes | 1.12(0.98, 1.28) | 0.083 |  |  |
| **Mental health care** |  |  |  |  |
| Access |  |  |  |  |
| No access | 1.8(1.20, 2.62) | **0.003** | 1.77(1.10, 2.75) | **0.014** |
| Abbreviations: CI = Confidence Interval, OR = Odds Ratio | | | | |
